# HOPX is required for the generation of umbilical cord blood-derived memory-like NK cells induced by three cytokines

**DOI:** 10.3389/fimmu.2026.1808687

**Published:** 2026-05-14

**Authors:** Zhiqing Liu, Yangyang Lei, Jiaxin Chen, Dan Mo, Xinyang Li, Xinrui Chen, Ruyu Liu, Dongmei Wang, Li Yan, Yan Ju, Yajing Huang, Changlin Yu, Huisheng Ai, Kaixun Hu, Mei Guo, Bo Cai, Yi Wang

**Affiliations:** 1 Senior Department of Hematology, Chinese PLA General Hospital, Beijing, China; 2 Graduate School of PLA Medical College, Chinese PLA General Hospital and PLA Medical College, Beijing, China; 3 The Affiliated Hospital of Chengde Medical University, Beijing, China; 4 Beijing Jishuitan Hospital, Capital Medical University, Beijing, China; 5 Department of Outpatient, Chinese PLA General Hospital, Beijing, China; 6 Senior Department of Obstetrics and Gynecology, Chinese PLA General Hospital, Beijing, China; 7 Innovvy (Beijing) Biomedical Technology Co. Ltd, Beijing, China

**Keywords:** anti-tumor, cytokines, HOPX, memory-like NK cells, umbilical cord blood

## Abstract

**Background:**

Natural killer (NK) cells represent a highly promising form of cancer immunotherapy. Recent studies have utilized umbilical cord blood (UCB) as a source of NK cells and achieved encouraging results. However, several challenges remain, including the limited number of NK cells that can be obtained from UCB, as well as the difficulty of ex vivo expansion and functional persistence, which hinder large−scale clinical applications. In addition, the absence of standardized culture systems leads to inconsistent cell purity and cytotoxic activity, thereby limiting the efficacy and translational potential of NK cell−based tumor therapy.

**Method:**

We demonstrated that *in vitro* activation by a combination of cytokines, followed by prolonged expansion with high doses of IL-2, can induce and expand memory-like NK cells from UCB. We performed both *in vivo* and *in vitro* investigations into the unique properties of these memory-like NK cells, and analyzed their heterogeneity via single-cell sequencing.

**Results:**

These memory-like NK cells displayed augmented proliferation and sustained cytotoxic efficacy. Via single-cell analysis, we detected considerable heterogeneity among UCB-derived NK cells. We identified six cell subsets with well-defined functional characteristics in expanded UCB-derived NK cells, conducted a systematic and in-depth analysis of the gene expression profile of each subset, obtained findings distinct from previous studies, and unraveled the unique transcriptional features of umbilical cord blood-derived NK cells. Moreover, memory-like NK cells exhibited a markedly higher proportion displaying a proliferative phenotype. Notably, we found the HOPX is required in the generation of memory-like NK cells derived from UCB.

**Conclusion:**

In conclusion, memory-like NK cells derived from umbilical cord blood exhibit excellent ex vivo expansion capacity and sustained cytotoxicity *in vitro* and *in vivo*. HOPX protein is required for the generation and functional maintenance of these cells. Therefore, such cells hold promising clinical potential in tumor immunotherapy.

## 1 Introduction

Natural killer (NK) cells are a crucial subset of innate lymphoid cells, and they play a significant role in defense against pathogens and anti-tumor immune responses (1, 2). While NK cell-based immunotherapies have not yet achieved the same success as adoptive T-cell therapies, increasing evidence from preclinical and clinical studies has demonstrated that they can serve as complementary cellular therapies alongside T-cell therapies (3). However, it is worth noting that the activation method, quantity, and source of NK cells directly impact the clinical outcome (4–7). To enhance the efficacy of NK cells, current research primarily focuses on two areas: optimizing the source of NK cells for adoptive transfer and improving their function and persistence within the host.

NK cells can be derived from various sources, such as peripheral blood (PB) (8), umbilical cord blood (UCB) (9), induced pluripotent stem cells (1), embryonic stem cells, et al (10). In peripheral blood, NK cells comprise approximately 10% of all lymphocytes, whereas in UCB, they can account for up to 30% of lymphocytes, making UCB a valuable source of NK cells (11). However, there are several challenges associated with the clinical use of NK cells derived from UCB. The UCB NK cells expansion efficiency without feeder cells *in vitro* is low (12, 13). Additionally, it is believed that UCB-derived NK cells are immature and exhibit weak cytotoxicity (14). These factors have limited the clinical utilization of NK cells derived from UCB.

To overcome the challenge of limited quantities of NK cells and improve their functionality, *in vitro* activation and expansion techniques have been employed. For expanding NK cells, two main approaches are commonly used: feeder cell methods and cytokine methods (15). Feeder cells, such as Epstein-Barr virus-transformed lymphoblastoid cell lines, mesenchymal stromal cells, genetically modified tumor cell lines expressing 4-1BB ligand and IL-21 are utilized for functional expansion and activation (16–18). Cytokine-induced stimulation of NK cells promotes their activation, particularly enhancing cytolytic activity and proliferation. In recent years, numerous studies have explored different expansion platforms for UCB NK cells. Large-scale expansion with cytokines like IL-2, IL-15, IL-21, and/or FLT-3 ligands can increase NK cell numbers (19–22). Nonetheless, this method has drawbacks, including limited cell expansion (23).

The aim of *in vitro* expansion is not only acquiring sufficient cell dose, but also pre-activating and modifying their antitumor features (24). Although NK cells have traditionally been considered innate immune system members, recent studies had identified NK cells with several immunological memory features following antigen exposure or viral infection. Memory NK cells can develop long-lasting and specific responses to different stimulatioins (25–30). Point-of-care memory-like NK cells were developed, and purified PB NK cells were stimulated with IL-12, IL-15, and IL-18 for 12 to 16 hours *in vitro*, also known as cytokine-induced memory-like (CIML) NK cells (31). Activated memory-like NK cells do not need to be expanded and were directly infused back into patients, which has achieved good clinical results (32–34). Although research on culture protocols for peripheral blood-derived memory-like NK cells is continually improving, the mechanism behind their potent cytotoxic and high proliferative ability remains unclear. On the other hand, comparing to peripheral blood NK cells, UCB NK cells showed distinct functions such as stronger proliferative capacity in response to cytokine stimulation, weaker cytotoxicity, and fewer adhesion molecules (9, 20). The ability of human UCB-derived memory-like NK cells to respond to cancer cells has not been reported, and limited information is available about the mechanisms involved in UCB-derived memory-like NK cell expansion. Meanwhile, NK cells exhibit certain heterogeneity, and in-depth analysis of different cell clusters’ functions is beneficial for the *in vitro* expansion of dominant cells. In this study, we found that expanded NK cells derived from UCB possessed the characteristics of memory-like cells, and their heterogeneity was investigated. Furthermore, UCB-derived memory-like NK cells were featured in high expression of the HOPX protein, and the expression level of HOPX was correlated with the cytotoxic and proliferative characteristics of NK cells.

## 2 Materials and methods

### 2.1 Cell lines and culture media

The K562, MCF-7, SW480, and HGC cell lines were purchased from American Type Culture Collection. All cell lines were cultured in Roswell Park Memorial Institute (RPMI,Invitrogen) medium supplemented with 10% fetal bovine serum (FBS,Invitrogen), 1% penicillin-streptomycin, 1% Lglutamine, 1% NEAA, and 1% sodium pyruvate.

### 2.2 NK cell purification and cell culture

Umbilical cord blood (UCB) samples were collected through the Beijing Cord Blood Bank. Normal donor PBMCs were obtained from anonymous healthy donors. All samples were isolated by Ficoll centrifugation. NK cells were purified by negative selection using EasySep Human NK Cell Enrichment kit (Stem Cell Technologies) with a purity of CD3^-^ CD56^+^ NK cells > 95%. To generate memory-like and control NK cells, purified NK cells were plated at 1-3x10^6^ cells/mL and preactivated for 12 hours using rhIL-12 (10 ng/mL,PeproTech) plus rhIL-18 (50 ng/mL,Biovision) and rhIL-15 (100 ng/mL,PeproTech) or control conditions(rhIL-2 1000TU/mL,PeproTech), washed 3 times to remove cytokines, and cultured in X-VIVO 15 (LONZA) medium containing 10% human AB serum(Geminibio), HEPES, NEAA, penicillin/streptomycin, and L-glutamine, supplemented with rhIL-2 (1000 TU/mL,PeproTech). Half of the medium was replaced every 2 days supplemented with rhIL-2. For the rhIL-15 cultured group, purified NK cells were preactivated and cultured with rhIL-15 (1 ng/mL,PeproTech).After being cultured for 14 days or the duration indicated in the text, NK cells were harvested, washed three times with PBS, and used for subsequent experiments.

### 2.3 HOPX KO in NK cell

We used CRISPR gene editing technology to genetically modify NK at the HOPX locus. The sequences and generation methods of the gRNAs were referenced from previously published literature (35). We cloned this gRNA into the lentiviral vector and packaged lentivirus using a three-plasmid system. Briefly, HEK293T cells were transfected with a mixture of pMD2.G, psPAX2, and the gene transfer plasmid via the calcium phosphate transfection method. Viruses packaged with empty vectors are used as the WT group. Two days post-transfection, the supernatants containing lentiviral particles were filtered and used for cell transduction. Following negative selection, NK cells were pre-stimulated with three cytokines for 12 hours. Prestimulated cells (1 × 10^6^) were resuspended in 2–3 mL of virus-conditioned medium supplemented with Polybrene (5 μg/mL, Sigma) in a 6-well plate,. The plates were centrifuged at 2500g for 90 minutes at 32 °C, followed by incubation at 37 °C for 3 hours. The multiplicity of infection (MOI) ranged from 5 to 30. After centrifugation, cells were incubated undisturbed for 24 hours in a humidified 37 °C, 5% CO_2_incubator. The transduction procedure was repeated on two consecutive days. Post-second transduction, cells were maintained in X-VIVO 15 (LONZA) medium supplemented with 10% human AB serum(Geminibio), and rhIL-2 (1000 TU/mL, PeproTech).

### 2.4 Flow cytometry analysis

Erythrocytes were removed by incubating with RBC lysis buffer (BD Biosciences) after UCB or peripheral blood cells were isolated by Ficoll density centrifugation. Isolated or expanded cells were stained in FACS buffer (1× PBS with 1% BSA) with specific antibodies for 20 min at 4 °C. Flow cytometric analysis was performed with the use of a BD FACS CantoII (BD Biosciences). The following monoclonal antibodies from Biolegend were used: anti-hCD56 PE (clone HCD56), anti-hCD3 FITC (clone SK7), anti-hCD16 APC/cy7 (clone 3G8), anti-hTIGIT APC (clone A15153G), anti-hNKG2D pe/cy7 (clone 1D11), anti-hPD-1 APC (clone A17188A), anti-hCD45 BV510 (clone 2D1), anti-hCD107a BV421 (clone H4A3), anti-mCD45 APC (clone 30-F11).

### 2.5 *In vitro* cytotoxicity assay

To investigate the killing capacity of ex vivo expanded NK cells, a FACS-based cytotoxicity assay was employed. The NK-sensitive HLA class I-negative cell line K562 was used as targets; 1-5x10^6^ target cells were labeled with 2uM cfse (invitrogen) for 15min at 37 °C. Cells were plated at 5x10^4^/well in 96-well-rounded bottom plates at effector to target (E:T) ratios for 4 h or 21h at 37 °C, 5% CO2. Cells were then stained with PE Annexin V Apoptosis Detection Kit with 7-AAD (Biolegend) in accordance with the manufacturer’s instructions and flow cytometry was performed. Dead/apoptotic cells within the cfse+ target cell population were considered lysed cells. Percent specific lysis was calculated using the following formula: % specific lysis=% experimental lysis−% basal lysis.

### 2.6 Real-time quantitative PCR

Total RNA was extracted using Trizol (Invitrogen) in accordance with the manufacturer’s instructions. cDNA synthesis performed with High Capacity cDNA Reverse Transcription Kit (Thermo Fisher Scientific) according to the manufacturer’s instructions. Real-time q-PCR was performed on LightCycler480 (Roche). Relative expression was determined by normalizing the amount of gene of interest to the GAPDH. Primer sets used in this study: HOPX (Fwd: 5′-tcaacaaggtcgacaagcac-3′; Rev: 5′-gtgacggatctgcactctga-3′), GAPDH (Fwd: 5′-gaaggtgaaggtcggagtc-3′; Rev: 5′-agatggtgatgggatttc-3′).

### 2.7 Adoptive transfer of NK cells into NPG mice

K562-luc was injected intravenously into NPG mice (6–10 weeks old). After 4 days, bioluminescence imaging (BLI) was performed to ensure leukemia engraftment, and control or memory-like NK cells were administered to the mice. The mice were treated with rhIL-2 every other day for 12 days and monitored or tumor burden (BLI) and survival. At indicated days, the mice blood were assessed for the presence of transferred cells by flow cytometry analysis. Mice were injected intraperitoneally with D-luciferin (150 mg/kg) (PerkinELmer) in PBS and imaged using the IVIS Lumina3 (PerkinELmer). BLI was performed at the indicated time points.

### 2.8 Single-cell RNA library preparation and sequencing

The cell suspension was loaded into Chromium microfluidic chips with 3’ (v2 or v3, depends on project) chemistry and barcoded with a 10× Chromium Controller (10X Genomics). RNA from the barcoded cells was subsequently reverse-transcribed and sequencing libraries constructed with reagents from a Chromium Single Cell 3’ v2 (v2 or v3, depends on project) reagent kit (10X Genomics) according to the manufacturer’s instructions. Sequencing was performed with Illumina (HiSeq 2000) according to the manufacturer’s instructions (Illumina).

### 2.9 scRNA-seq data processing

Raw reads of FASTQ format were de-multiplexed and converted using Illumina bcl2fastq software. The cDNA insert was mapped to the GRCm38 reference genome. Only confidently mapped, non-PCR duplicates with valid barcodes and unique molecular identifiers were used to generate the gene-barcode matrix. All downstream single-cell analyses were performed using Seurat R package73. A gene with expression in more than 5 cells was considered as expressed and each cell was required to have at least 1,000 expressed genes.

### 2.10 Cell type annotation

We merged and analyzed all samples using the Seurat package in R for clustering and result visualization. First, we used the LogNormalize function to normalize all gene expression data and the FindVariableFeatures function to automatically select the top 2000 features. After standardizing the data, we selected the PCA principal component analysis method to reduce the dimensionality of the data, used UMAP to map the high-dimensional spatial data to a low-dimensional space, and visualized the data. We also used the SeuratFindClusters function in the R package to identify clusters and divide all cells into 6 clusters.

### 2.11 Cluster marker identification

First, we used the SingleR package to automatically correlate the gene expression profiles of clusters in the data with those in the reference library. The package will annotate the clusters automatically based on the ranking of correlation with each cluster, assigning the cell types with the highest relevance to each cluster. Then, we used the FindMarkers function in the Seurat package to analyze the highly variable genes in each cluster. After obtaining the characteristic marker genes for each group, plot them as dot plots. Name and validate the clusters based on previously reported related genes in the literature, and finally obtain the names of the clusters.

### 2.12 Cell cycle and pseudotime analysis

We used the CellCycleScoring package to predict the G1, S, and G2M phases. The cell cycle phase of each cell was determined based on whether the G1/S/G2M gene set was highly expressed. The score for each phase was calculated as the ratio of the number of highly expressed marker genes to the total number of marker genes. The corresponding score represented the degree of high expression of the gene set for that phase in the cell, thus predicting the cell’s phase in the cycle.

Using the Monocle2 package, all cells were subjected to pseudotime trajectory analysis (https://github.com/cole-trapnell-lab/monocle-release). The Differential GeneTest function in Monocle2 was used to identify genes differentially expressed under different developmental conditions. A non-linear reconstruction algorithm, DDR tree, was used to construct a pseudotime trajectory with the top 1000 genes with the lowest q-values, showing the distribution of cells in various groups on the pseudotime tree.

### 2.13 Differential gene expression and KEGG enrichment analysis

To identify differentially expressed genes between different groups, the FindAllMarkers function was executed in Seurat with parameters (log2 FC. Threshold = 0.25, test. Use = Student’s t-test). Differential genes were compared using the Wilcoxon test, where P<0.05 and fold change >1.5 were defined as differentially expressed genes between the two comparison groups. Based on the compared differential gene sets, enrichment pathways were obtained through Kyoto Encyclopedia of Genes and Genomes (KEGG). Pathways with P.adj<0.05 were considered as significant enrichment pathways, and the Benjamin-Hochberg statistical method was used to estimate the false discovery rate (FDR).

### 2.14 Statistical analysis

Statistical analyses and graph generation were performed using GraphPad Prism 9(GraphPad Software Inc., San Diego, CA, USA). Data are presented as mean± SEM. For conventional physiological data, normality and homogeneity of variance were assessed using the Shapiro–Wilk and Levene’s tests, respectively. One-way analysis of variance (ANOVA) and Tukey’s *post hoc* test were employed to perform statistical analyses. Additionally, an unpaired t-test was used to compare the difference between two groups.

## 3 Results

### 3.1 The combination of cytokines IL-12, IL-15, and IL-18 greatly enhanced the expansion efficiency of NK cells derived from UCB

**Figure 1:**
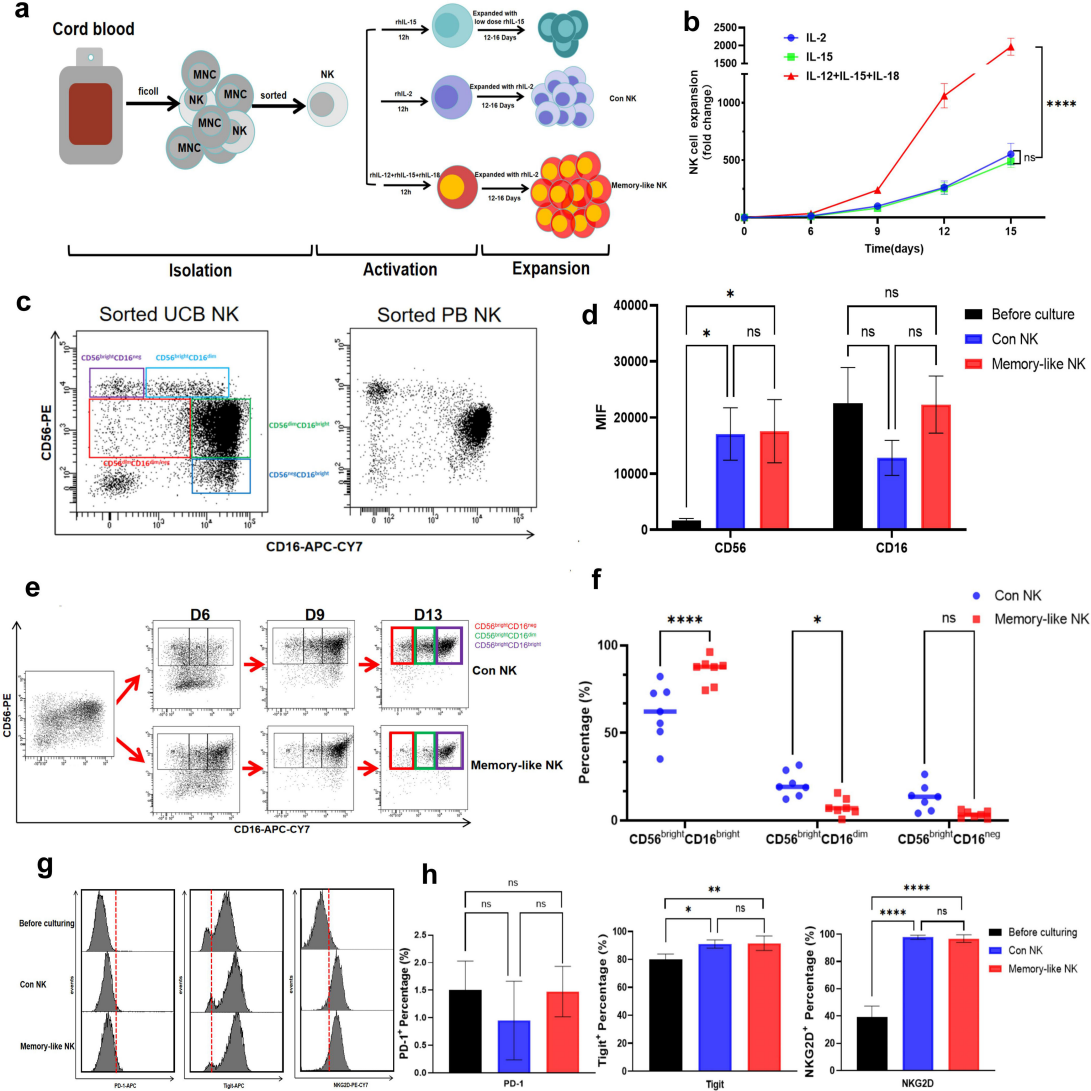
UCB-derived memory-like NK cells exhibit enhanced proliferation capacity and specific phenotypes. **(a)** Schema of *in vitro* expansion protocol of NK cells derived from umbilical cord blood. **(b)** Counting results of NK cells from the same donor expanded under different protocols for 15 days. Memory-like NK cell amplification was significantly better than the other groups(n = 6 from three independent experiments). **(c)** Representative flow plots showing each typical NK cell subsets of sorted NK cells from UCB or peripheral blood. **(d)** Summary of data showing the changes in mean fluorescence intensity (MFI) of CD16+ and CD56+ before and after expansion. **(e)** Representative flow plots showing the changes of each subpopulation of sorted cord blood under different culture protocols for 13 days. **(f)** Summary of data from **(e)** showing the difference in the proportion of CD56brightCD16dim, CD56brightCD16neg cells in the two culture protocols(n = 7 from three independent experiments). **(g)** The representative flow histogram showed the expression of immune checkpoint molecules Tigit, PD-1, and activating molecule NKG2D before and after culture. **(h)** Summary of data from **(g)**. Tigit and NKG2D expression increased significantly after culture(n = 4 from two independent experiments). ns - not significant, *P value < 0.05, **P value < 0.01, ****P value < 0.0001 determined by two-way ANOVA **(b)**, one-way ANOVA with Tukey’s multiple comparisons **(d, h)** or unpaired two-tailed t test **(f)**.

Mononuclear cells (MNC) were isolated from UCB cells using Ficoll-Paque solution, and then NK cells were sorted using negative selection. The purity of CD3^-^ cells after sorting was more than 95% (**Supplementary Figure 1A**). Initially, the control group was treated with either IL-15 or IL-2 alone, while the experimental group received a combination of IL-12, IL-15, and IL-18 (tri-combination). The sorted NK cells were activated for 12 hours, after which all cytokines were removed. Subsequently, the cells were further expanded using either IL-15 or IL-2 for a period of 12 to 16 days, as shown in **Figure 1A**. We observed a significant variation in the expansion of NK cells after 15 days with different cytokine regimens. The combination of the three cytokines resulted in a 1968-fold expansion (range, 1719-fold to 2300-fold), whereas IL-2 or IL-15 alone led to expansions of 553-fold (range, 421-fold to 668-fold) and 509-fold (range, 428-fold to 579-fold), respectively. However, the difference between the two groups was not statistically significant (**Figure 1B**). Peripheral blood NK cells were also successfully expanded using IL-15 alone, and the amplification fold was even better than with IL-2 alone (36). However, we observed a potential risk of failure when culturing UCB-derived NK cells with IL-15 alone (four out of seven cultures were successful). This suggests that NK cells from different sources may have distinct requirements for culture methods. For subsequent experiments, we used the group cultured with IL-2 alone as control.

### 3.2 Tri-combination-activating UCB NK cells showed distinct phenotypes after culture

Most molecular profiling studies pertaining to human NK cells have primarily focused on the comparison between CD56^bright^ and CD56^dim^ NK cells. CD56^bright^ NK cells are commonly regarded as the precursors of CD56^dim^ NK cells, which are considered to be immature (37). During maturation, the expression of CD56 on NK cells gradually decreases while CD16 expression gradually increases. It has been well established that CD56^bright^ and CD56^dim^ NK cell clusters differ in terms of their functionality. CD56^dim^CD16^+^ NK cells (CD56^dim^ NK cells) possess cytotoxic capabilities and can directly eliminate target cells, whereas CD56^bright^CD16^-/low^ NK cells (CD56^bright^ NK cells) are primarily responsible for cytokines production (38). Numerous studies have reported the presence of a substantial number of immature NK cells in cord blood. Similarly, we discovered that uncultured UCB NK cells can be classified into five clusters: CD56^bright^CD16^neg^, CD56^bright^CD16^dim^, CD56^dim^CD16^bright^, CD56^neg^CD16^bright^, and CD56^dim^CD16^dim/neg^, which included the atypical CD56^neg^CD16^bright^ population in peripheral blood (**Figure 1C**) (9). We observed that throughout the culture process, the mean fluorescence intensity of CD56 significantly increased under both culture protocols. However, there was no difference in the mean fluorescence intensity of CD16 between the two protocols before and after culture (**Figure 1D**). After 14 days of culture, cells in both groups could be categorized into three clusters: CD56^bright^CD16^bright^, CD56^bright^CD16^dim^, and CD56^bright^CD16^neg^ (**Figure 1E**). Interestingly, the proportion of CD56^bright^CD16^bright^ NK cells in the memory-like group accounted for 87.6% (range 74.4% to 96.2%), with only a few other clusters of NK cells present. Conversely, the average proportion of CD56^bright^CD16^bright^ NK cells in the control group was only 62.2% (range 45.1% to 82.2%). The percentages of CD56^bright^CD16^dim^ and CD56^bright^CD16^neg^ cells also exhibited considerable differences between two groups (5.9% vs. 14.1% and 2.4% vs. 10.5%, respectively) (**Figure 1F**). Additionally, the molecules Tigit, PD-1, and NKG2D were detected on NK cells before and after culture (**Figure 1G**). Results demonstrated that the proportion of PD-1^+^ NK cells was consistently low both before and after culture, with no discernible difference between control and memory-like groups. However, the average fluorescence intensity of PD-1 molecules was significantly enhanced after culture, with no difference seen between two groups. Correspondingly, the proportion of TIGIT^+^ NK cells remained high before and after culture, and both culture methods were found to significantly increase this proportion. Furthermore, the mean fluorescence intensity of TIGIT molecules exhibited considerable increase under both culture methods, with no difference observed between the two protocols. On the other hand, only a small proportion of NK cells expressed NKG2D before culture. However, the proportion of NKG2D^+^ cells increased significantly after culture, with no difference observed between the two culture protocols (**Figure 1H**, **Supplementary Figure 1B**).

### 3.3 UCB-derived memory-like NK cells exhibited sustained cytotoxicity *in vitro*

**Figure 2:**
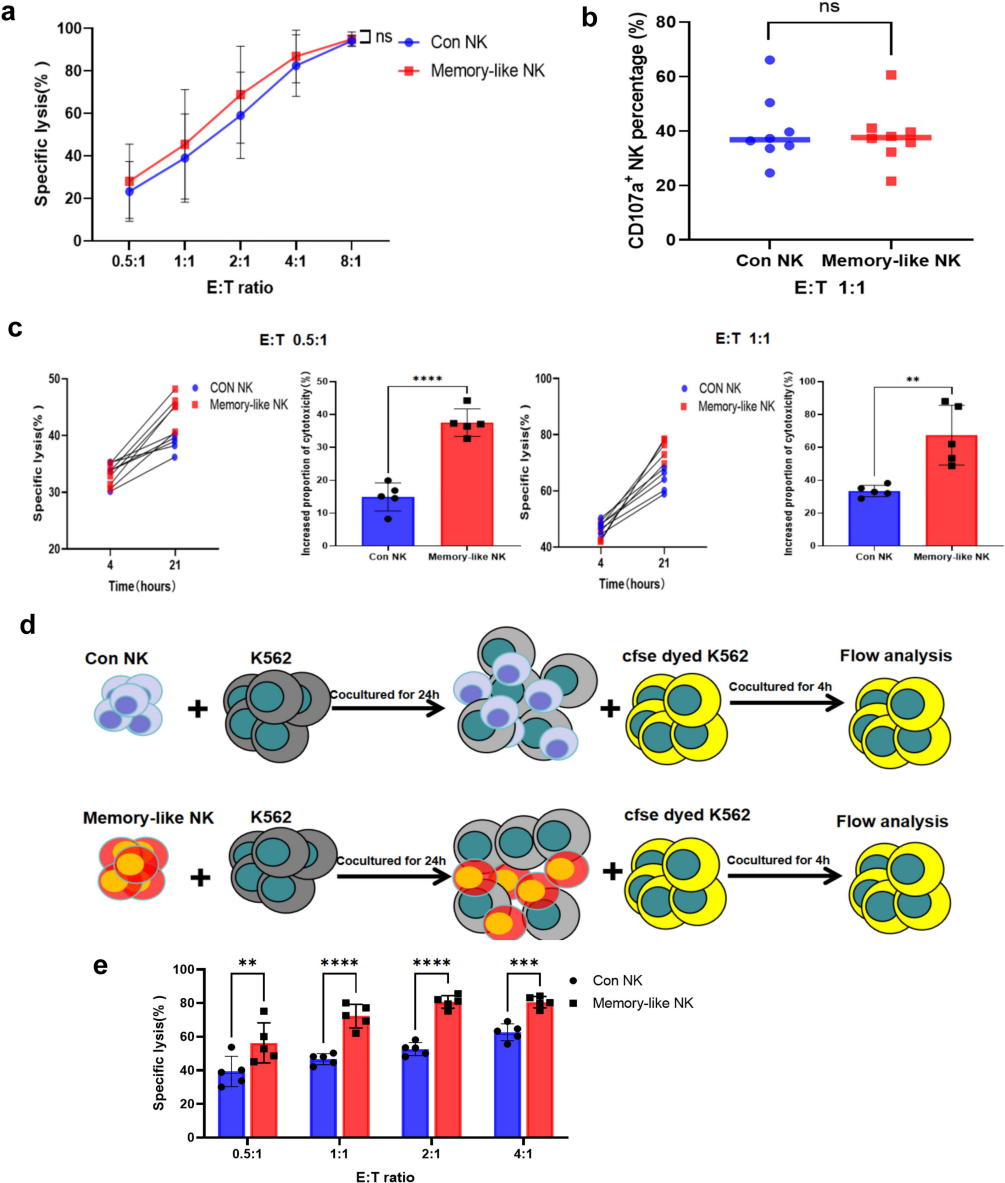
UCB-derived memory-like NK cells exhibit sustained functional responses against leukemia targets. **(a)** The cytotoxicity of NK cells in two groups against K562 tumor cells at different effector-target ratios(n = 8 from three independent experiments). **(b)** The expression of CD107a in two groups (n = 8 from three independent experiments). **(c)**The apoptosis of tumor cells after co-incubation with tumor cells for 4 hours and 21 hours at the effector/target ratios of0.5:1 and 1:1 (n = 5 from two independent experiments). **(d)** Schema of secondary killing test. **(e)** Secondary killing and apoptosis of tumor cells in the two groups under different effector/target ratios(n = 5 from two independent experiments). ns, not significant, *P value < 0.05, **P value < 0.01, ****P value < 0.0001 determined by two-way ANOVA **(a)** or unpaired two-tailed t test **(b, c, e)**.

Previous studies have demonstrated that memory-like NK cells derived from peripheral blood exhibit enhanced cytotoxic effects. Therefore, it is important to investigate whether NK cells derived from UCB possess similar functions. In our study, when NK cells were co-cultured with tumor cells for 4 hours at different effector/target (E/T) ratios, we observed that, unlike the results from previous studies on peripheral blood-derived cells, there were no differences in the cytotoxic effects of the two groups of cells at the same E/T ratio (**Figure 2A**). Furthermore, the expression of CD107a in NK cells was also similar (**Figure 2B**). However, intriguingly, when the killing time was extended to 21 hours, differences became evident. At the low E/T ratios of 0.5:1 and 1:1, the tumor cell death rate in the memory-like group increased by 33.47% and 67.43%, respectively, whereas in the control group it only increased by 14.93% and 37.55% (**Figure 2C**). At an E/T ratio of 2:1, both groups achieved killing rates exceeding 90%. In contrast, four hours was insufficient for both groups to achieve this killing rate, requiring an E/T ratio greater than 4:1 (**Supplementary Figure 1C**). To further evaluate the sustained killing ability of NK cells, we re-cultured NK cells that had been co-cultured with tumor cells for 21 hours with new tumor cells (**Figure 2D**). Remarkably, we found that the cytotoxic effect of memory-like NK cells was significantly stronger than that of NK cells in the control group (**Figure 2E**).

**Figure 3:**
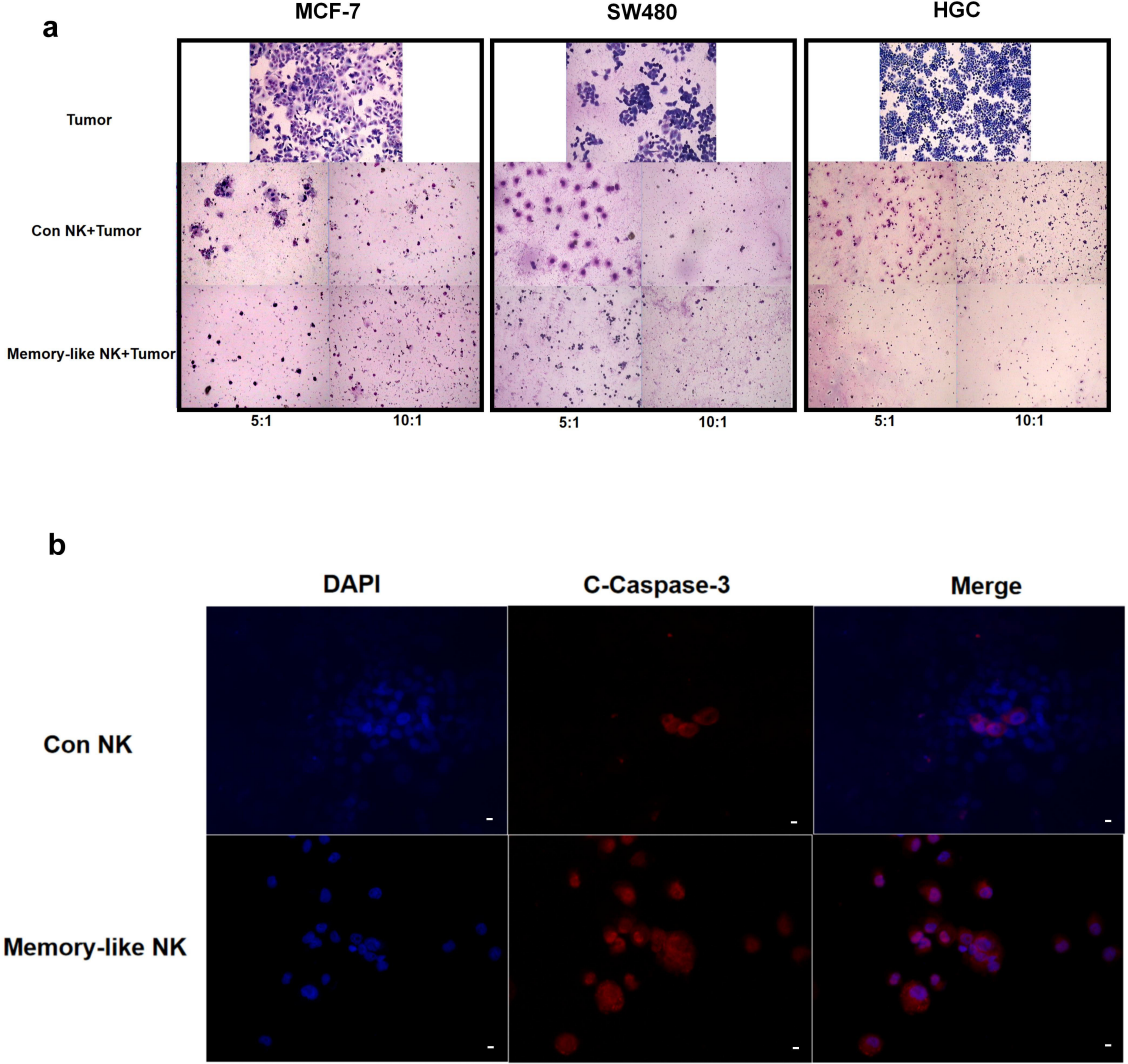
UCB-derived memory-like NK cells exhibit stronger cytotoxicity against solid tumor cells. **(a)** HE staining showed that the two groups of NK cells had cytotoxic effects on solid tumor cell lines MCF-7, SW480, and HGC at the effector/target ratio of 5:1 and 10:1 after co-incubation for 24 hours. The memory-like NK cells showed stronger cytotoxicity. **(b)** The results of immunofluorescence showed that the two groups of NK cells could activate the Caspase-3 signaling pathway after co-incubation with SW480 cells for 4 hours, and the effect of memory-like NK cells was more obvious. Scale bar, 10 um.

In addition to utilizing the sensitive cell line K562 as target cells, we conducted further investigations on the cytotoxic effect of UCB-derived memory-like NK cells on solid tumor cell lines. Specifically, we employed the adenocarcinoma cell line MCF-7, colorectal cancer cell line SW480, and gastric cancer cell line HGC as target cells. Our findings demonstrated that UCB-derived NK cells exhibited a killing effect on these solid tumor cell lines. Moreover, the memory-like NK cells also displayed enhanced cytotoxicity (**Figure 3A**). Additional analysis revealed that NK cells induced apoptosis of tumor cells through the Caspase3 signaling pathway (**Figure 3B**).

### 3.4 UCB-derived memory-like NK cells showed longer survival *in vivo* and prolonged the survival of tumor-bearing mice

**Figure 4:**
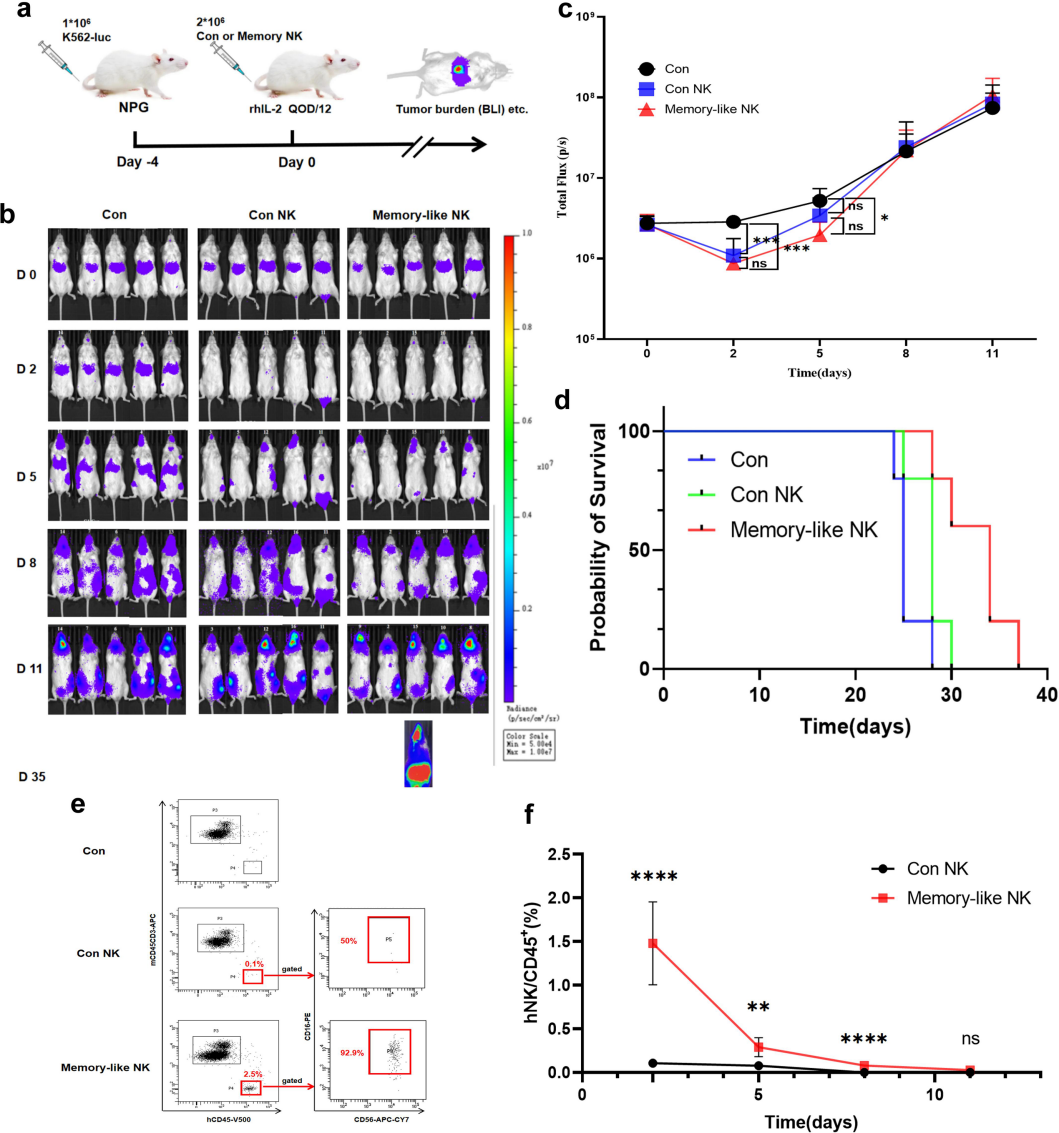
*In vivo* results show that UCB-derived memory-like NK cells exhibit sustained functional responses against leukemia targets. **(a)** Schema of *in vivo* experiments. Experimental design for **(b-f)**. K562-luc was injected intravenously into NPG mice. After 4 days, BLI was performed to ensure leukemia engraftment, and control or memory like NK cells were administered to the mice. The mice were treated with rhIL-2 every other day for 12 days and monitored for tumor burden (BLI) and survival. **(b)** Representative BLI of recipient mice engrafted with K562-luc on the indicated day after tumor administration. **(c)** Summary of serial BLI measurements that show reduced tumor burden in mice receiving memory-like NK cells compared to control NK cells on day 2 and day 5. **(d)** Mice were treated as in **(a)**, monitored for survival, and analyzed using the log-rank test. **(e)** Representative flow plots showing the survival of NK cells in the peripheral blood of mice at different time points after infusion. **(f)** Summary of data showing that the cells in the memory group could be continuously detected in the periphery of the mice on day 2, 5, and 8 after infusion, and were significantly higher than those in the control group, which could not be detected on day 8 after infusion. ns, not significant, *P value > 0.05, **P value < 0.01, ****P value < 0.0001 determined by one way ANOVA with Tukey’s multiple comparisons **(c)**, unpaired two-tailed t test **(f)**, or log-rank test **(d)**.

Furthermore, we explored whether UCB-derived memory-like NK cells exhibited prolonged survival time and enhanced cytotoxicity *in vivo*. NPG mice were injected with luciferase gene-transfected K562 tumor cells. Four days later, imaging revealed the presence of tumor cells in mice. Subsequently, mice were divided into three groups, and those from two groups were injected with control and memory-like NK cells, respectively, at a low ratio of 2:1 via the tail vein. Mice from the other group were injected with PBS (the blank group). This injection was considered day 0. To evaluate NK cell survival, NK cells were injected only once, while IL-2 was injected every other day to support NK cell survival (**Figure 4A**). At day 2, minimal tumor cells were observed in the two groups injected with NK cells, whereas a large number of tumor cells were present in the abdomen of mice that did not receive NK cells. At day 5, the fluorescence intensity of tumor cells in the memory-like NK group was lower than that in the non-injected group, while no difference was observed between the injected control group and the non-injected group. By day 8, the fluorescence intensity of tumor cells in all three groups had become consistent (**Figures 4B, C**). However, the introduction of memory-like NK cells extended the survival of mice, with median survival times of 25, 28, and 34 days, respectively, in blank, control NK, and memory-like NK groups (**Figure 4D**). Furthermore, at day 2, the proportion of control NK cells in the peripheral blood was only one-twelfth of that of memory-like NK cells. By day 8, conventional NK cells could not be detected in the peripheral blood, while memory-like NK cells were still detectable until day 11. The survival time of memory-like NK cells derived from cord blood was significantly longer than that of conventional NK cells (**Figures 4E, F**, **Supplementary Figure 1D**).

### 3.5 Single-cell transcriptomics of UCB-derived NK cells

**Figure 5:**
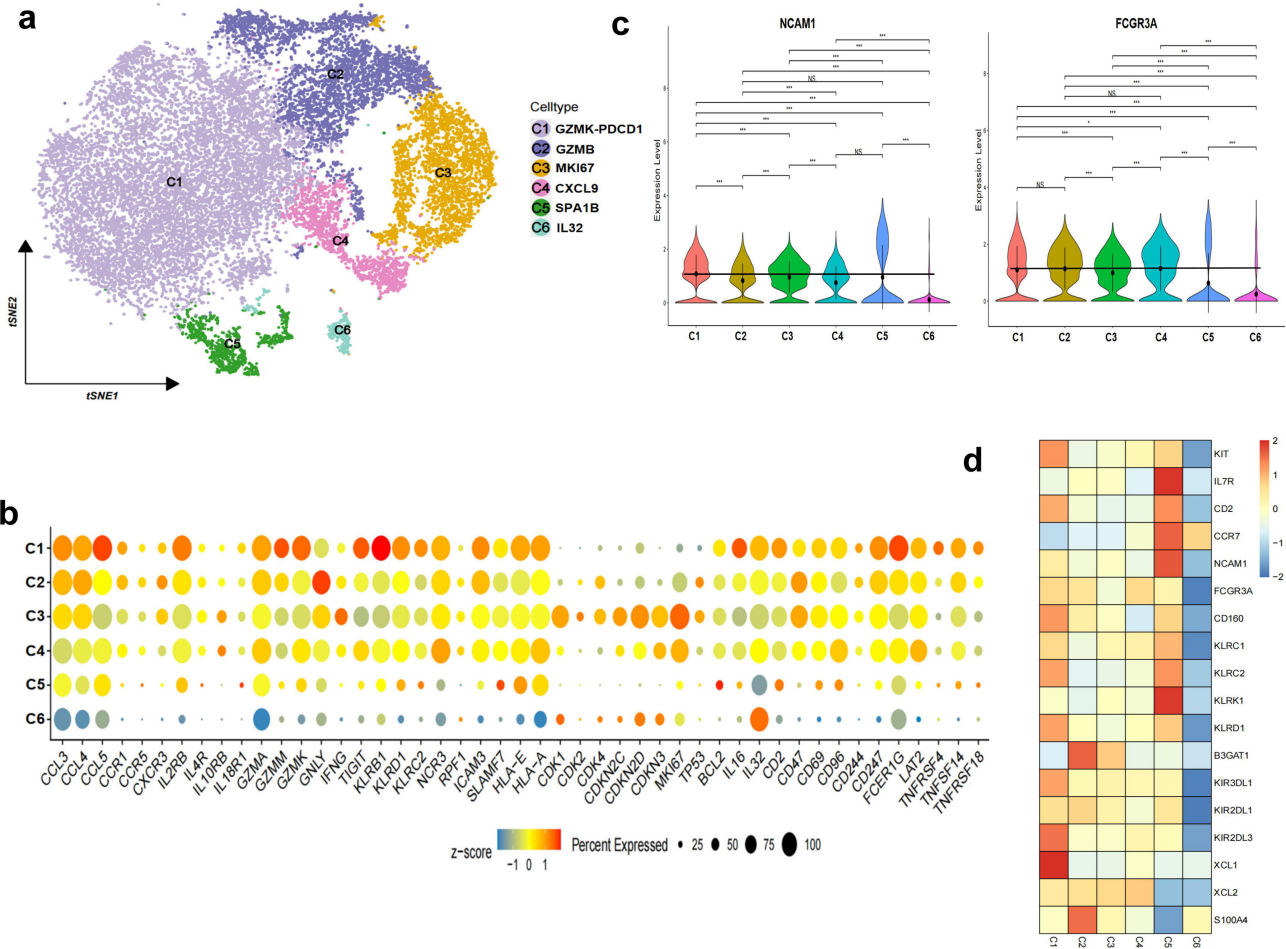
Transcriptomic landscape of the expanded UCB-derived NK cells. **(a)** Uniform manifold approximation and projection (UMAP) visualization of main lineages of the NK cells based on single-cell transcriptomes. Each dot represents a single cell; colors indicate cell clusters with numbered labels. **(b)** Heatmap showing the expression pattern of selected signature genes of the NK cell clusters. Dot size represents the fraction of expressing cells. Color indicates the Z score scaled gene expression levels. **(c)** Violin plots showing the expression pattern of *NCAM1* and *FCGR3A*. **(d)** Heatmap showing the expression pattern of typical genes with maturation of the NK cells. *P value < 0.05, **P value < 0.01, ****P value < 0.0001.

To comprehensively analyze the transcriptome characteristics and population heterogeneity of UCB-derived memory-like NK cells, we performed single-cell transcriptome sequencing on two groups of cells. We obtained NK cells from cord blood using different culture protocols from two donors. After strict quality control, we obtained a total of 21,289 cells. Based on the highly variable gene expression in each cell, we divided all cells into six clusters (**Figure 5A**; **Supplementary Figure 2A**). Clusters 1, 2, and 3 represented the largest populations, accounting for 55%, 18%, and 14% of the total cells, respectively (**Supplementary Figure 2B**). Previous studies have indicated that cord blood NK cells are mostly immature cells. Therefore, we hypothesized that *in vitro* culture would induce the generation of NK cells with varying degrees of maturity. Specifically, the C1-GZMK-*PDCD1* cluster predominantly contained terminal mature NK cells, which highly expressed genes related to cytotoxicity. Additionally, this cluster showed high expression of immune checkpoint molecules, including *PDCD1*. The C2-*GZMB* subset referred to mature NK cells, as it highly expressed genes related to cytotoxicity and DNA synthesis. The C3-*MKI67* cluster mainly contained NK cells with high proliferation ability, exhibiting high expression of cell cycle-related proteins and *MKI67*. The C4-*CXCL9* cluster primarily consisted of transitional NK cells, expressing characteristic genes at an intermediate level. The C5-*HSPA1B* cluster mainly contained activated mature NK cells, and demonstrated high expression of molecules related to stress response. Conversely, the C6 cluster hardly expressed any other characteristic genes, apart from *IL-32* (**Figure 5B**). C1, C2, and C3 groups expressed more chemokines and cytokines, including *CCL4, CCL3, CCL5, XCL2, CCL4L2, CCL3L3, IL-32, IL-16, TNF*, and *IFNG* (**Supplementary Figure 2C**).

Previous studies have identified the involvement of NK cell clusters in various developmental stages, as evidenced by their expression of lineage-specific genes. NK cells undergo progressive development, transitioning from CD56^bright^ to CD56^dim^ NK cells, which is accompanied by the expression of specific receptors associated with distinct maturation states (39). For instance, immature NK cells exhibit high expression of *KIT, IL7RA, CD56, CD62L, NKG2A*, and granzyme K (*GZMK*). In mature clusters, there is notable upregulation of molecules crucial for cytotoxic function, including perforin, granzymes, CD16, CD57, and members of the killer immunoglobulin-like receptor (*KIR*) family (40). However, our findings indicate that the expression of several molecules in most subpopulations yielded contradictory results compared to *in vivo* development. For instance, the expression of *NCAM1* (encoding CD56) and *FCGR3A* (encoding CD16) did not exhibit a reciprocal trend but rather tended to be co-expressed (**Figure 5C**). Additionally, the strong expression of *KIT* and *IL7RA* was not synchronized, with *KIT* being highly expressed in C1 while *IL7RA* was predominantly expressed in C5. Combining the high expression of mature NK cell surface marker *CD160* and the *KIR* family in C1, we speculated that it was in the mature stage. However, the difference was that it also highly expressed immature markers such as *KIT, KLRC1*, and *GZMK*, especially low expression of mature killer NK cell surface marker CD57 (*B3GAT1*). C2 cells, with low expression of *CD160* and the killer *KIR* family but high expression of CD57 (*B3GAT1*), together with high granzyme expression, were also assumed to be mature (**Figure 5D**). However, none of these states were typical of homeostatic cells. These results suggest that there are variations in the developmental patterns between our cultured UCB-derived NK cells and the homeostatic NK cells *in vivo*.

### 3.6 UCB-derived NK cells showed distinct population characteristics in the expression of killing and proliferation-related molecules

**Figure 6:**
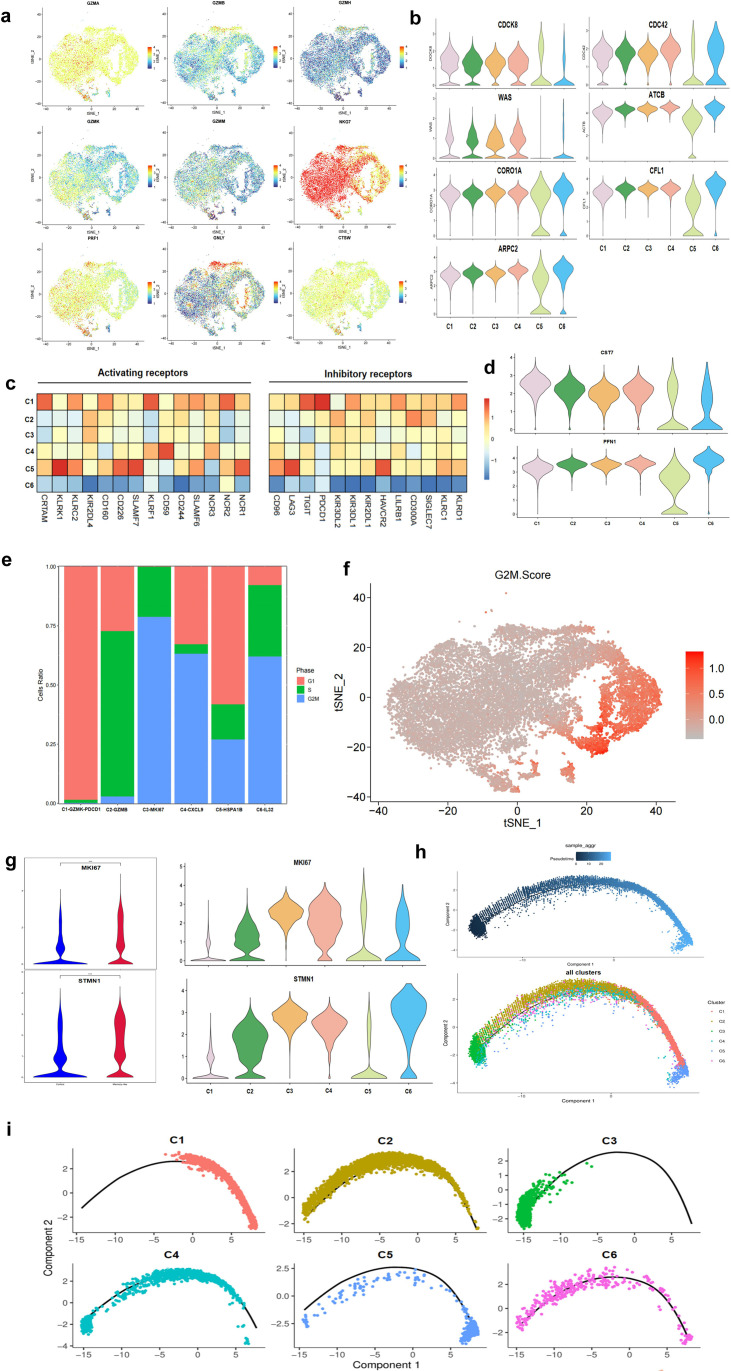
Characteristics of UCB-derived NK cells in the expression of killing and proliferation-related molecules. **(a)** tSNE plot showing the expression of cytotoxic effector genes. Expression is color coded from blue (low) to red (high). **(b)** Violin plots showing the expression pattern of molecules involved in cytoskeletal remodeling of the NK cell clusters. **(c)** Heatmaps showing the inhibitory and activating receptors expression pattern in NK cell clusters. **(d)** The expression of *CST7 and PFN1* of the six clusters were shown in violin plots. **(e)** Stacked bar chart showing the constitution of different cell cycle phases of six clusters expression of G1, S, and G2/M gene sets. **(f)** tSNE plot showing the G2/M score of the NK cells. Expression is color coded from gray (low) to red (high). **(g)** Violin plot showing the expression of *MK167 and STMN1* in control and memory-like NK cells and six subsets of the two groups **(h)** Pseudotime trajectory of cultured UCB-derived NK cells with pseudotime. **(i)** Pseudotime trajectory of individual cluster demonstrated in the trajectory.

Mature NK cells are cytotoxic cells capable of directly killing target cells. It is not surprising that resting NK cells, which respond quickly to stimulation, have higher expression of genes encoding effector molecules. Analysis of cytotoxic-related genes revealed that *GZMA, GZMM, GZMK, PRF1*, and *NKG7* were highly expressed in C1, while *GZMB, GZMH*, and *GNLY* were highly expressed in cluster C2. C4 and C5 expressed some killer effector molecules at a low intensity, and C6 expressed all effector molecules at an even lower intensity (**Figure 6A**). Interestingly, several sequencing studies have indicated that CD56^hi^ immature NK cell clusters highly express *GZMK*, which differs from T cells, in which high *GZMK* expression is associated with cell exhaustion. Optimal cytotoxicity requires cytoskeletal remodeling (41). However, we found that C1 and part of C5 only expressed cytoskeletal remodeling-related molecule *DOCK8* at a high intensity, while cells in C4 and C6 expressed related molecules such as *CORO1A, CFL1*, and *CDC42* at high levels (**Figure 6B**).

The main regulation of NK cells is the balance between surface activating receptors and inhibitory receptors (42). Previous studies have shown that CD94/NKG2A, NKp46, NKp44, CD160, and NKG2D are variably expressed in different maturation states, while CD16 and multiple KIRs are markers of mature NK clusters, and CD57 indicates terminal differentiation (40). Our findings reveal that the C1 expressed high levels of most activation molecules, such as NCRs (NKp46, NKp44, NKp30) and the costimulatory molecule *KLRF1* (encoding NKp80). The C5 showed high expression of *CD226* (DNAM-1), NKp46, *KLRK1* (encoding NKG2D), and *KLRC2* (encoding NKG2C). The C4 expressed moderate levels of most activation molecules. The other clusters exhibited low levels of activating molecules, especially the C6 cluster, which only expressed NKp44. In terms of inhibitory receptors, the C1 and C5 also highly expressed *KLRD1 (CD94), KLRC1 (NKG2A)*, and other inhibitory molecules. Additionally, the C2 expressed the *CD300A* molecule. NK cells may express additional immune checkpoints, including PDCD1, TIGIT, TIM-3, LAG-3, and CD96 (43). We found that the C1 strongly expressed PDCD1, while the C5 strongly expressed *LAG-3* and *HAVCR2* (encoding TIM-3). However, the expression of these immune checkpoints in other clusters was not high (**Figure 6C**). It has been reported that the expression of these immune checkpoints is upregulated in tumor-associated NK cells in different malignancies. Therefore, it has been hypothesized that these immune checkpoints, mainly TIGIT, could play a role in carcinogenesis by inhibiting NK cell cytotoxicity. Additionally, the C1 highly expressed the negative regulatory protein *CST7*, but did not show high expression of another negative regulatory protein *PFN1*. In contrast, the C5 cluster exhibited low expression of both proteins (**Figure 6D**). In summary, C1 and C5 had similar expression trends for many molecules, but the types of expression differed. Furthermore, the C5 cluster expressed numerous stress-related molecules and the immediate early genes (IEGs) category, including *NR4A2, DUSP1, FOSB, FOS, JUN*, and *JUNB* (**Supplementary Figure 2D**). This suggests that C5 subset cells were a population of activated mature NK cells, despite having the highest intensity of CD56 molecules (44, 45).

The results of our cell cycle analysis showed that the C1 and C5 predominantly resided in the G0/G1 phase. The majority of cells in C2 were in the S phase, while clusters C3, C4, and C5 consisted mainly of cells in the G2/M phase (**Figures 6E, F**). Furthermore, our KEGG enrichment analysis revealed significant enrichment for DNA synthesis and cell cycle signaling pathways in clusters C2, C3, and C4 (**Supplementary Figure 2E**). These three clusters exhibited a high level of proliferative activity, particularly C3 and C4. Furthermore, we observed elevated expression levels of *TP53* and *MKI67* molecules in these two clusters (**Figure 6G**).

The advantage of scRNA-seq analysis lies in its capability to model the developmental order in a population with heterogeneous development, based on transcriptional changes observed during cell differentiation. In this study, we employed quasi-temporal trajectory analysis to characterize the developmental order of various clusters. Our findings revealed that most of the cells in the C1, along with all of the cells in the C5, were situated at one end of the developmental trajectory. Furthermore, the C5 was positioned closer to the edge of the trajectory compared to the C1. Conversely, the C3 was located at the other end of the developmental trajectory. Integrating the results obtained from pseudo-time analysis with those from our previous analysis, we concluded that the C3 represented the least mature subgroup among the clusters, while the C1 and C5 represented the final stages of development (**Figure 6H**). Several other clusters were distributed along the trajectory, with the C2 primarily occupying the anterior and middle segments of the trajectory, the C4 mainly presenting in the anterior and posterior segment, the C6 cluster predominantly distributing in the anterior segment (**Figure 6I**).

### 3.7 The characteristics of UCB-derived memory-like NK cells

**Figure 7:**
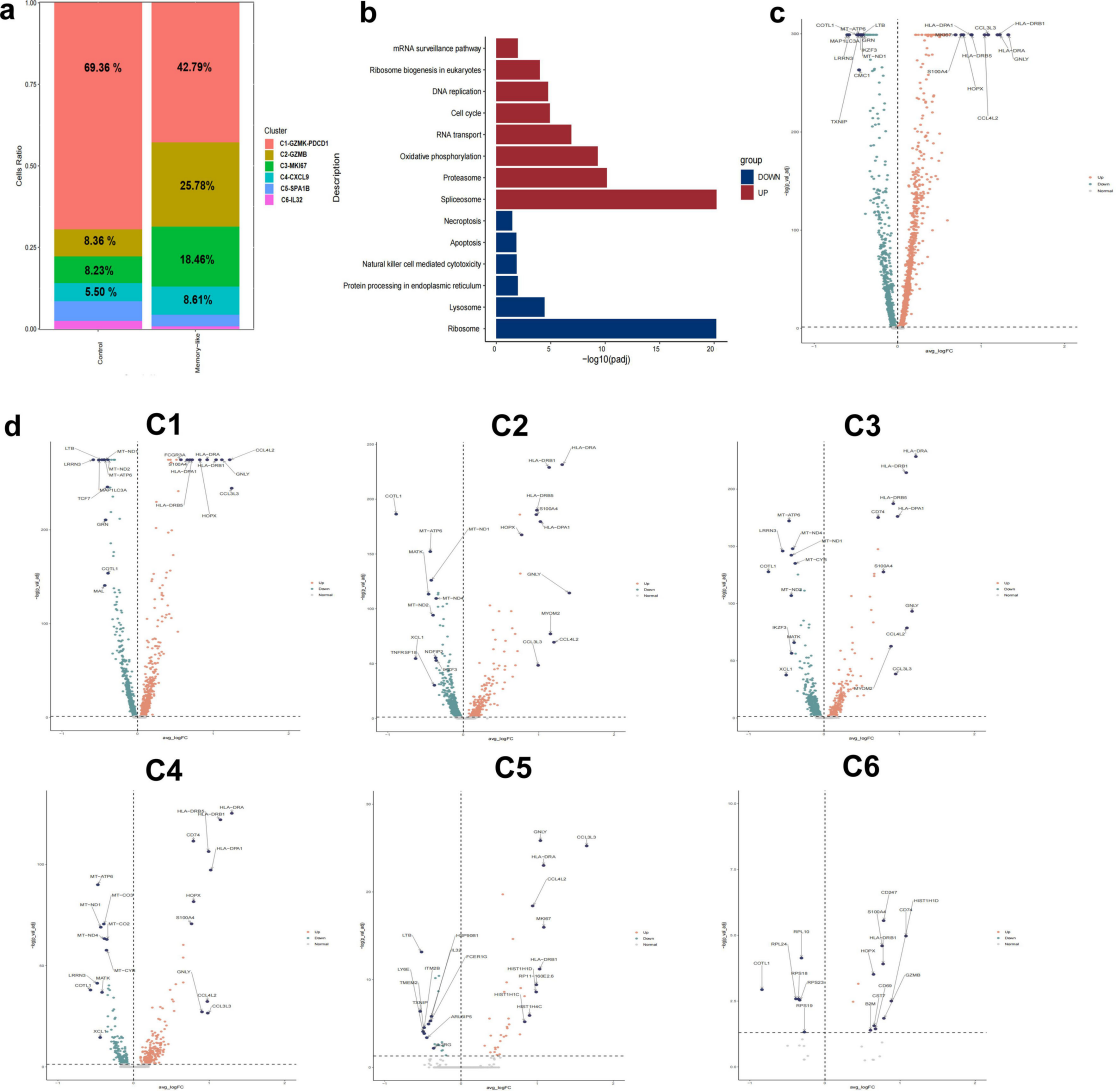
Differences in the transcriptome of memory-like and control NK cells. **(a)** The proportion of the six groups of NK cells in the control and memory clusters. **(b)** Bar plots from the Metascape analysis showing the major enriched terms of DEGs of the two groups. The length of each bar represents -log10 (padj value). **(c)** Volcano plot showing DEGs of memory-like and control NK cells. DEGs were detected by differential expression analysis (twosided Student’s t-test). Each dot represents a single gene, the ascending and descending top ten geneswere both indicated. **(d)** Volcano plot showing DEGs of each cluster of memory-like and control NK cells. DEGs were detected by differential expression analysis (two-sided Student’s t-test). Each dot represents a single gene, the ascending and descending top ten genes were both indicated.

In our previous *in vitro* and *in vivo* studies, we observed differences in the proliferation and survival capabilities between two groups of NK cells. We aimed to uncover the distinction at the single-cell level. Although both groups exhibited all types of NK cells, their proportions differed significantly. The control NK cell group demonstrated proportions of 69.36%, 8.36%, 8.23%, 5.50%, 6.04%, and 2.51% for the six cell clusters, respectively. Conversely, the memory-like NK cell group displayed proportions of 42.79%, 25.78%, 18.46%, 8.61%, 3.56%, and 0.80%, respectively (**Figure 7A**). The proportions of clusters C2, C3, and C4 notably increased in memory-like cells, while the proportions of clusters C1, C5, and C6 significantly decreased. Thus, the robust proliferation ability of C2, C3, and C4 clusters could explain the sustained killing capacity of memory cells both *in vitro* and *in vivo*.

We conducted further analysis on the differential genes expressed in cells cultured in two different ways. Our findings showed that the number of up-regulated genes in memory-like NK cells, when compared to control NK cells, was 1574, while the number of down-regulated genes was 812. When performing KEGG analyses on the differentially expressed genes (DEGs), we observed that the up-regulated DEGs in memory-like NK cells were mainly enriched in spliceosome, proteasome, oxidative phosphorylation, cell cycle, and DNA replication pathways. Conversely, the down-regulated genes were primarily enriched in ribosome and lysosome related signaling pathways (**Figure 7B**).

Heatmaps were generated by selecting the top 30 genes with the most significant p-values for each putative cluster between the two groups (**Supplementary Figure 3A**). KEGG analyses on the differentially expressed genes (DEGs) for clusters were performed (**Supplementary Figure 3B**). For further analysis, genes that increased or decreased were selected to create a volcano plot. The top 10 genes with increased expression mainly including *GNLY, HLA-DRs, CCL4L2, CCL3L3, HOPX, S100A4* and *MKI67*. And the top 10 genes with decreased expression mainly including *MAP1LC3A, LRRN3, COTL1, TXNIP, MT-ATP6, CMC1, IKZF3, MT-ND1, LTB* and *GRN*(**Figure 7C**).The increased expression genes in each clusters were concentrated (**Figure 7D**).GNLY was the molecule whose expression was upregulated in most clusters of memory-like NK cells. GNLY is a cytolytic molecule expressed by both human CTL and NK cells, which exhibits activity against various tumors and microbes (46, 47). S100A4 increases with NK cell maturation, with highest expression seen on mature NK cells (48). HLA-DR is considered as a marker of late activation. Previous research has demonstrated that HLA-DR^+^ NK cells exhibit functional activity, such as producing proinflammatory cytokines, degranulating, and easily proliferating in response to stimuli. Furthermore, HLA-DR^+^ NK cells appear capable of capturing and presenting specific antigens to CD4^+^ and CD8^+^ T cells, thereby inducing their activation and proliferation (49–51). *CCL4L2* and *CCL3L3* were the upregulated proinflammatory chemokine genes in C1-C4 clusters of memory-like NK cells, which were associated with viral infections and autoimmune diseases. Among the genes with downregulated expression in the C1 of memory-like NK cells, *GRN* (*Granulin*) and *TCF7* (*Transcription Factor 7*) are particularly significant. Progranulin (PGRN, encoded by the *GRN* gene) can interferes with NK cell activation and expansion (52, 53). TCF7 is required for the establishment and maintenance of memory T cells (54). In a similar fashion, TCF7 was shown to be required for the establishment and maintenance of memory NK cells when virus infection (55). The *IKZF3* gene is downregulated in the C2 and C3 of memory-like NK cells. IKZF3 can affect ILC1/NK cell transdifferentiation and function (56). In the C5 of memory-like NK cells, the expression of the *TXNIP* gene is downregulated. TXNIP can promote human NK cell differentiation by affecting protein synthesis and proliferation of early NK cell differentiation stages, but it is redundant for functional NK cell maturation (57).

### 3.8 The HOPX protein is highly expressed in UCB-derived memory-like cells

**Figure 8:**
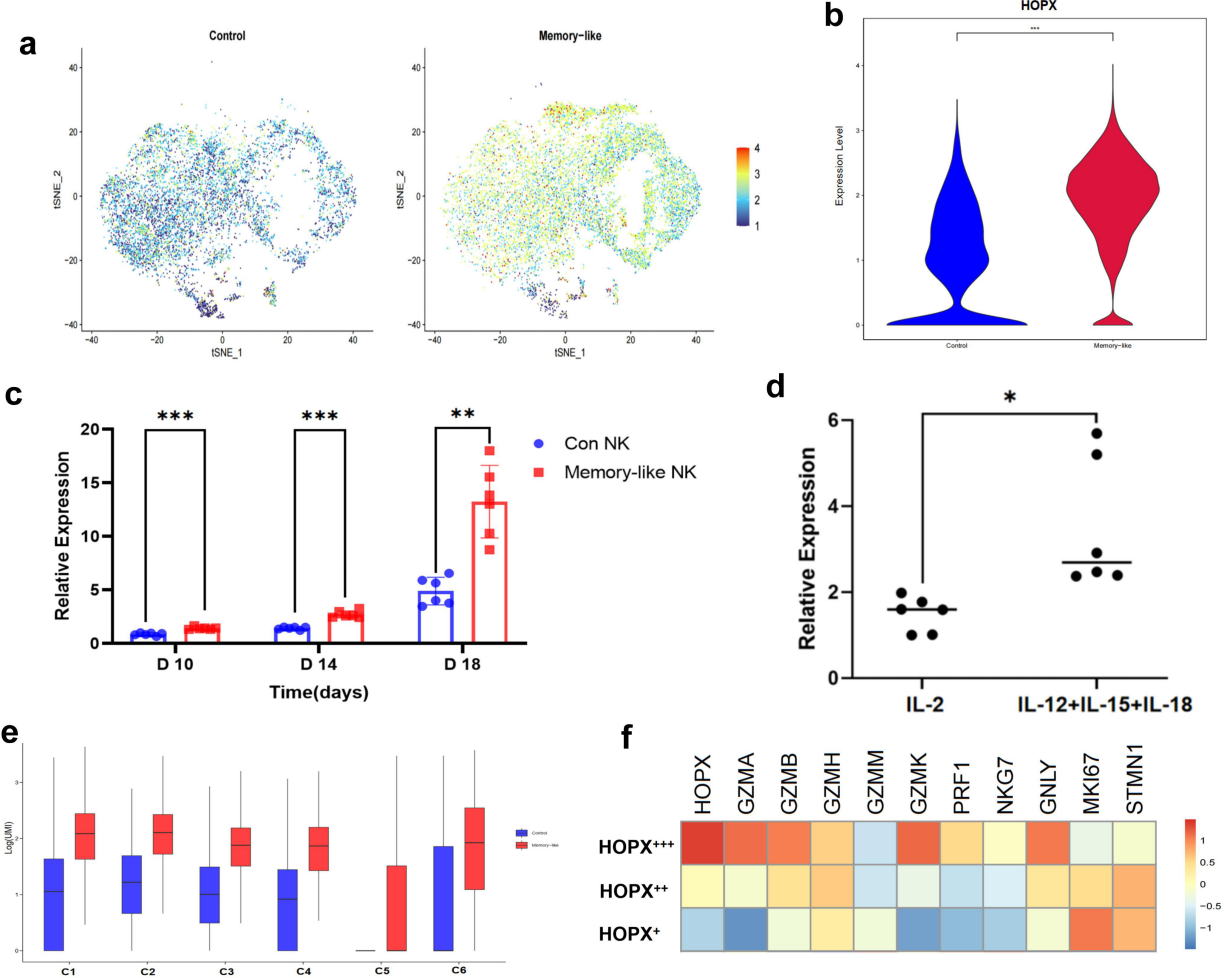
The difference in the expression of the *HOPX* between the control NK group and the memory-like NK group. **(a)** tSNE plot showing the expression of *HOPX* genes of memory-like and control NK cells, respectively. Expression is color coded from blue (low) to red (high). **(b)** Violin plot showing the expression of *HOPX* genes of memory-like and control NK cells. **(c)** Real-time PCR was used to detect the expression changes of *HOPX* gene with the expansion of NK cells from the UCB in the control group and the memory-like group(n = 6 from three independent experiments). **(d)** Changes in the relative expression of *HOPX* gene in peripheral blood-derived NK cells through different expansion processes(n = 6 from three independent experiments). **(e)** The *HOPX* gene expression of the six clusters of the control and memory-like groups. **(f)** Heatmaps showing the expression of cytotoxic molecules and proliferation-related proteins of the three subgroups derived from the memory-like NK cells according to the expression of *HOPX* genes.*P value < 0.05, **P value < 0.01, ****P value < 0.0001 determined by unpaired two-tailed t test **(c, d)**.

HOPX is upregulated in induced regulatory T cells and effector memory T cells and is critical for the survival of activated mouse T helper type 1 effector-memory cells (58, 59). HOPX is also overexpressed in memory NK cells, suggesting that it may promote the persistence of memory NK cells (60). But whether HOPX is expressed similarly in memory-like NK cells and its relationship with the generation of memory properties remain unclear in current research. We found that *HOPX* was mainly expressed in UCB-derived memory-like cells (**Figures 8A, B**). We validated our experimental findings using fluorescence quantitative PCR and observed consistent results, noting a gradual increase in expression levels with prolonged culture time (**Figure 8C**). Similarly, when culturing peripheral blood-derived NK cells using the same method, activation by the three-cytokine combination, along with long-term culture and expansion, led to significantly elevated expression of HOPX (**Figure 8D**). Upon further exploring the expression profiles of individual clusters, we observed markedly higher expression levels across all clusters in the memory-like group compared to the control group. Notably, within both groups of cells, subpopulation C2 exhibited the highest expression, followed by C1 and then C3. Furthermore, based on *HOPX* expression levels, we stratified memory-like NK cells into three subgroups: HOPX^+++^, HOPX^++^, and HOPX^+^ subgroups. Interestingly, even within the *HOPX* low expression subgroup, the average expression of *HOPX* in the memory-like NK group surpassed that of the control NK group. Through gene analysis, we discovered a positive correlation between the expression levels of cytotoxic effector molecules (with the exception of *GZMM*) and *HOPX* in memory-like NK cells. Conversely, the expression levels of proliferation-related proteins and cell viability exhibited an inverse correlation with *HOPX* expression levels (**Figure 8F**).

### 3.9 The HOPX protein is required in maintaining the characteristics of UCB-derived memory-like cells

**Figure 9:**
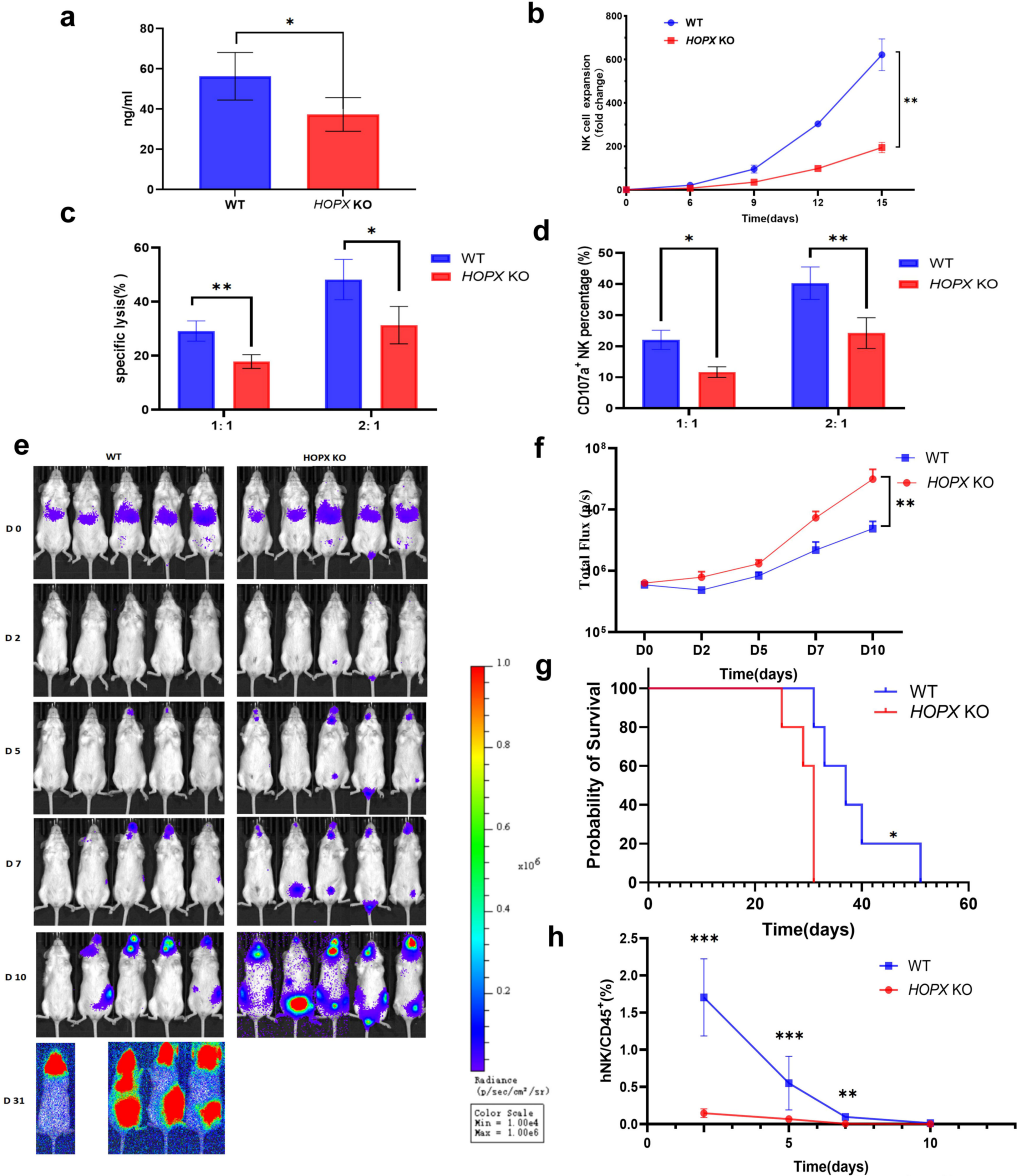
The effect of the HOPX on the properties of memory-like NK cells. **(a)** The expression of HOPX in WT vs *HOPX* KO groups(n = 6 from three independent experiments). **(b)** The effect of *HOPX* gene editing on the expansion of NK cells *in vitro*(n = 6 from three independent experiments). **(c)** The killing effect of NK cells from WT group and *HOPX* group on K562 cells at effector-target ratios of 1:1 and 2:1(n = 6 from three independent experiments). **(d)** The expression of CD107a in NK cells from WT group and *HOPX* KO group against K562 cells at effector-target ratios of 1:1 and 2:1(n = 6 from three independent experiments). **(e)** Representative BLI of recipient mice engrafted with K562-luc on the indicated day after tumor administration. **(f)** Summary of serial BLI measurements that show reduced tumor burden in mice receiving memory-like NK cells compared to *HOPX* KO NK cells. **(g)** Mice were treated as in **(e)**, monitored for survival, and analyzed using the log-rank test. **(h)** Summary of data showing that the cells in the memory group could be continuously detected in the periphery of the mice on day 2, 5, and 7 after infusion, and were significantly higher than those in the *HOPX* KO group. ns - not significant, *P value 0.05, **P value < 0.01, ****P value < 0.0001 determined by unpaired two-tailed t test **(a, c, d, h)**, two-way ANOVA with Tukey’s multiple comparisons **(b, f)** or log-rank test **(g)**.

Next, to investigate the role of HOPX in the generation of memory-like NK cells derived from UCB induced by three cytokines, we employed CRISPR/Cas9 to knockout the *HOPX* gene in NK cells prior to culture (**Figure 9A**). Notably, the reduction in HOPX expression significantly attenuated the *in vitro* proliferative capacity of NK cells (**Figure 9B**). More critically, both the cytotoxic activity of NK cells against K562 cells and their degranulation function were markedly diminished (**Figures 9C, D**). At the animal level, we infused wild-type (WT) and *HOPX* knockout (KO) memory-like NK cells into mice inoculated with K562 cells. Imaging results showed that on the second day after NK cell infusion, both groups effectively eliminated K562 cells. However, as time progressed, the tumor burden in mice of the *HOPX* KO group were significantly higher than that in the WT group (**Figures 9E, F**). Naturally, *HOPX* KO significantly shortened the survival time of mice (**Figure 9G**). Through tracking the infused NK cells in mice, we found that *HOPX* KO significantly reduced the proportion of infused NK cells in the mice (**Figure 9H**). Collectively, these findings demonstrate that *HOPX* gene knockout profoundly impairs the acquisition of memory-like functions in umbilical cord blood-derived NK cells.

## 4 Discussion

NK cells are critical components of innate immunity, performing essential functions in combating foreign pathogens and eradicating transformed cells. NK cell-based immunotherapy holds significant promise in cancer treatment (61). However, clinical research on NK cells has also revealed limitations, including their inability to undergo sustained *in vivo* expansion and their lack of persistence (15). To enhance their effectiveness, larger quantities of NK cells are required.

A brief *in vitro* stimulation of NK cells with interleukin (IL)-12, IL-15, and IL-18 endow them a memory-like behavior (32). In this study, NK cells derived from UCB were cultured in the presence of IL-12, IL-15, and IL-18. The combination of these three cytokines led to a significant expansion of NK cells, compared to the use of IL-2 alone. Prior to culture, UCB-derived NK cells could be classified into five clusters: CD56^bright^CD16^neg^, CD56^bright^CD16^dim^, CD56^dim^CD16^bright^, CD56^neg^CD16^bright^, and CD56^dim^CD16^dim/neg^. After the culture process, the cells maintained a certain level of heterogeneity, primarily comprising CD56^bright^CD16^bright^, CD56^bright^CD16^dim^, and CD56^bright^CD16^neg^ populations. Notably, the combination treatment with the three cytokines resulted in a higher abundance of CD56^bright^CD16^bright^ cells. Importantly, the combined activation of these three factors significantly enhanced the anti-tumor activity and survival time of NK cells against K562 cells *in vivo*, indicating the development of memory-like NK cells. Moreover, the memory-like NK cells exhibited enhanced cytotoxicity against various solid tumor cell lines and induced apoptosis in tumor cells through the Caspase3 signaling pathway.

We employed scRNA-sequencing technology to investigate the heterogeneity of UCB-derived memory-like NK cells. Cells were divided into six clusters, with C1-C3 accounting for over 80% of the population. We observed an evolutionary order among the subpopulations. Additionally, we noted that the phenotype of UCB-derived NK cells cultured *in vitro* differed from that of NK cells in homeostasis. Classifying the maturation status of these cells using commonly used surface markers proved challenging, as contradictory expression patterns were observed, indicating unique phenotypic characteristics.

Specifically, we identified the C1 as a mature terminal group, characterized by high expression of cytotoxic molecules. Interestingly, high expression of certain activating molecules coincided with increased levels of inhibitory molecules in C1 cells. Moreover, these cells demonstrated minimal proliferative capacity. C2 cells exhibited high expression of some cytotoxic molecules, but the levels of activating and inhibitory molecules were not as pronounced. This subgroup displayed robust proliferation and was classified as mature NK cells. C3 cells expressed cytotoxic molecules at moderate levels, while the expression of activating and inhibitory molecules was relatively low. C3 cells showed the highest proliferation ability and were considered immature NK cells. Similarly, C4 NK cells expressed cytotoxic markers at moderate intensity, with relatively low levels of activating and inhibitory molecules. C4 cells exhibited strong proliferation capacity and were categorized as transitional NK cells. The C5 subgroup displayed moderate expression of cytotoxic markers, high expression of certain activating molecules, and simultaneously high expression of inhibitory molecules. Notably, the proliferation capacity of C5 cells was lower compared to C3 and C4. Moreover, the high expression of stress-related genes suggested that these cells represented an activated subset of mature NK cells. Lastly, C6 cells exhibited low expression levels of the aforementioned molecules, but demonstrated high expression of *IL-32*. Considering the simulated time distribution trajectory, we hypothesized that C6 cells were in the early stages of development.

Additionally, we observed a significant increase in the proportion of C2, C3, and C4 in the group of memory-like NK cells derived from UCB. This finding provides some insight into the enhanced expansion capacity and prolonged survival time of memory NK cells. Through differential gene analysis, we also identified expression changes in *HLA-DR, GNLY, CCL4L2, CCL3L3*, and other molecules.

High expression of the HOPX required was observed in memory-like group cells, which is of utmost importance. *HOPX* is a homeobox gene that encodes the smallest known member of the homeodomain-containing protein family, a homeodomain-only protein (62, 63). Unlike typical homeobox proteins, HOPX does not directly bind to DNA to regulate gene expression. Instead, it acts as a co-factor by binding to various protein partners, including serum response factor (SRF), histone deacetylases (HDACs), GATA4, and potentially other factors. This binding allows HOPX to recruit transcription factors to gene promoter regions and regulate molecular mechanisms. One of the functions of HOPX is to modulate the expression of immediate-early genes, such as *Jun* and *Fos*, which play a role in cell proliferation and differentiation (64, 65). However, conflicting data from different studies have reported that HOPX can either promote or inhibit different molecular pathways, making it a critical factor in maintaining the balance between cellular proliferation and differentiation. Early induction of HOPX expression has been associated with stemness and differentiation trajectories in various non-hematopoietic progenitor populations, including cardiomyocyte-committed cardiac progenitor cells, keratinocytes, adult intestinal stem cells, colitis-associated regenerative stem cells, et al (66–69). Previous studies have indicated that high expression of HOPX contributes to the survival of memory T cells and memory NK cells (70).Our findings confirm that the combination of three cytokines can effectively induce the generation of memory-like NK cells derived from UCB, with HOPX playing an important role in this process. Furthermore, HOPX shows great potential as a characteristic marker for cytokine-induced UCB-derived memory-like NK cells. In summary, our study revealed the biological characteristics of UCB-derived memory-like NK cells at the single-cell level, facilitating future investigations into adoptive therapy utilizing UCB-derived NK cells.

## Data Availability

The datasets presented in this study can be found in online repositories. The names of the repositories and accession numbers can be found in the **Supplementary Material**.
