# Supplementary figures and images for: HOPX is required for the generation of umbilical cord blood-derived memory-like NK cells induced by three cytokines
Source: Front Immunol. 2026 May 14;17:1808687. doi: 10.3389/fimmu.2026.1808687 (PMC13215855; doi:10.3389/fimmu.2026.1808687)

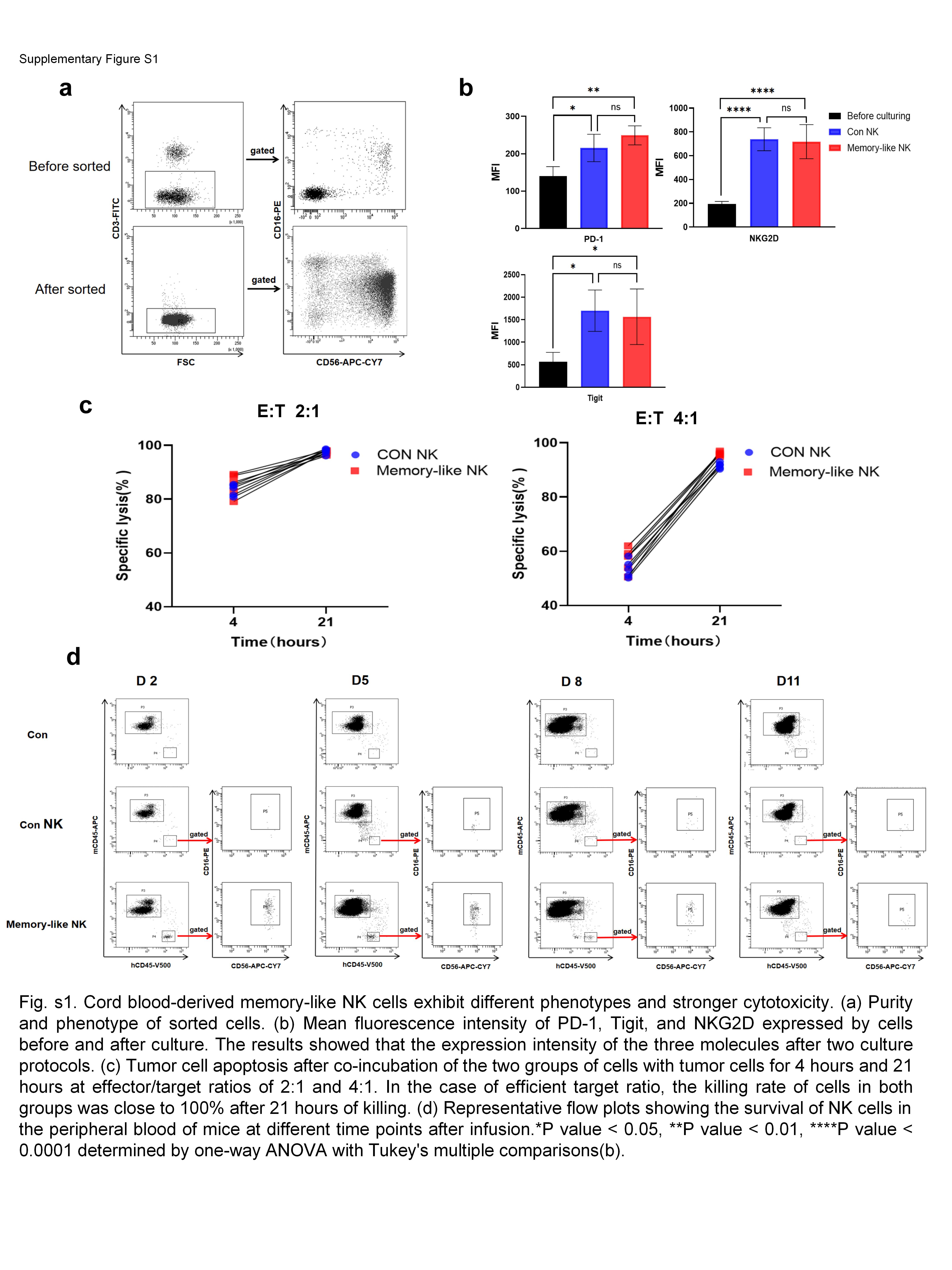

Supplement: Supplementary file 1 [file Image1.jpg]

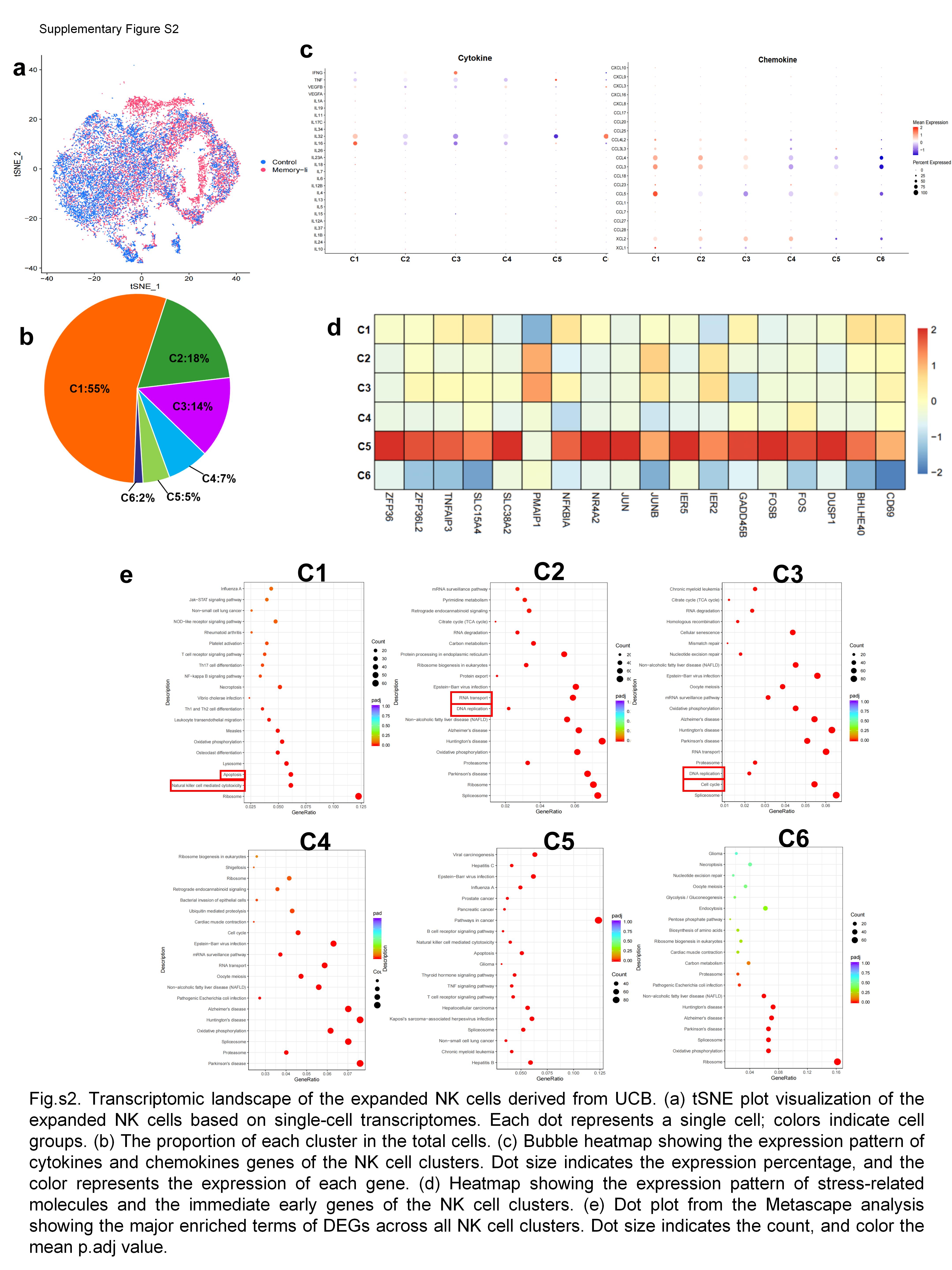

Supplement: Supplementary file 2 [file Image2.jpg]

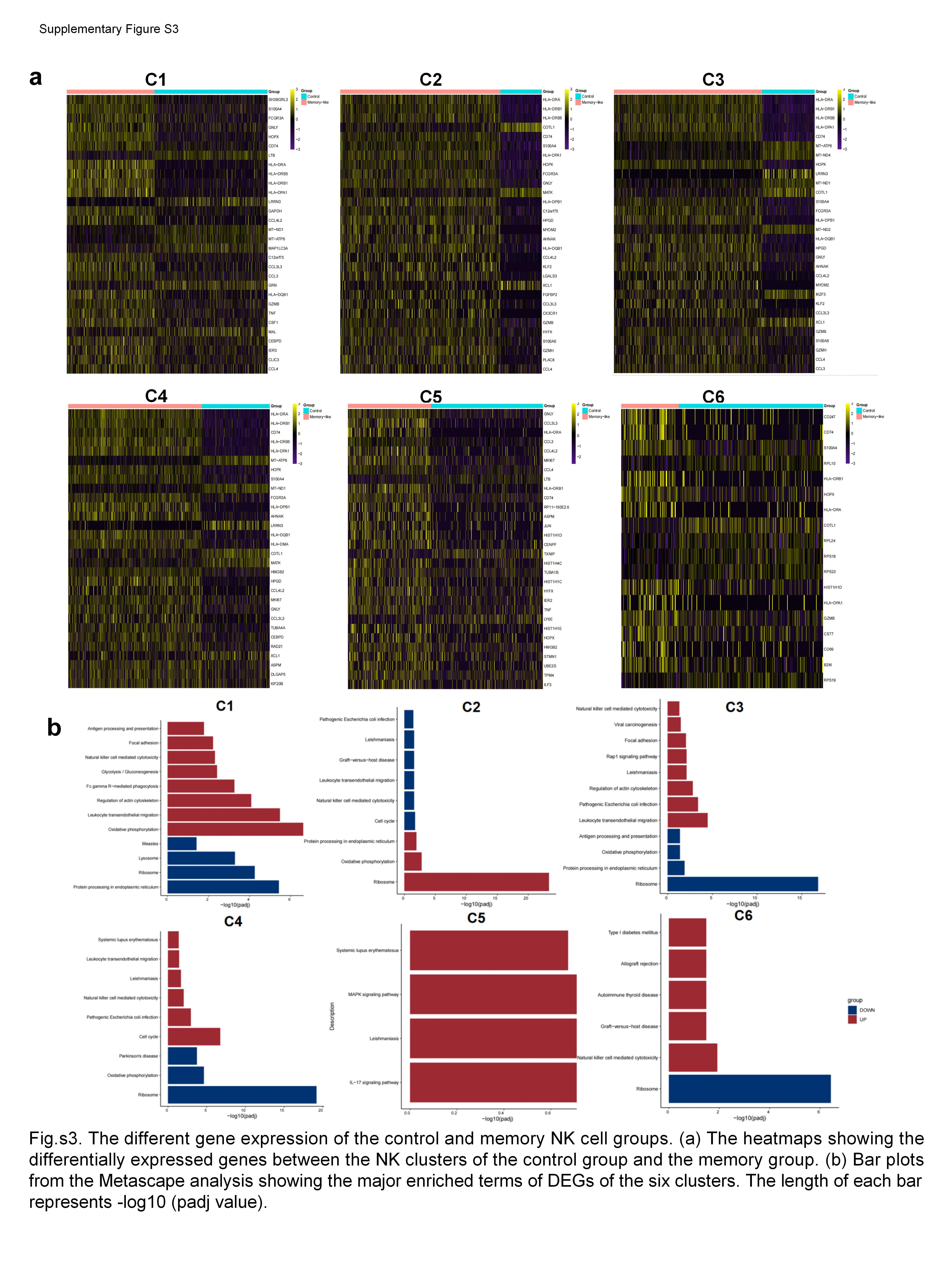

Supplement: Supplementary file 3 [file Image3.jpeg]
